# Critical illness and bone metabolism: where are we now and what is next?

**DOI:** 10.1186/s40001-022-00805-w

**Published:** 2022-09-14

**Authors:** Yun Cai, Fuxin Kang, Xiaozhi Wang

**Affiliations:** Department of Critical Care Medicine, the Second Affiliated Hospital of Hainan Medical College, Hainan province Haikou City, China

**Keywords:** Critical illness, Bone metabolism, Osteoporosis, Nutritional support, Rehabilitation training

## Abstract

Critical illness refers to the clinical signs of severe, variable and life-threatening critical conditions, often accompanied by insufficiency or failure of one or more organs. Bone health of critically ill patients is severely affected during and after ICU admission. Therefore, clinical work should focus on ICU-related bone loss, and early development and implementation of related prevention and treatment strategies: optimized and personalized nutritional support (high-quality protein, trace elements and intestinal prebiotics) and appropriate physiotherapy and muscle training should be implemented as early as possible after ICU admission and discharge. At the same time, the drug regulates excessive metabolism and resists osteoporosis.

## Introduction

Critical illness is defined as ‘‘clinical signs of severe, variable, and life-threatening critical conditions, often accompanied by insufficiency or failure of one or more organs ‘‘ [1]. Despite extensive research efforts, many challenges remain in assessing short- and long-term prognosis in critically ill patients (CIPs) [2]. Critically ill survivors still face higher mortality, physical and cognitive impairment, and psychological distress compared with premorbid state and general population control subjects. The physical domain mainly refers to muscle weakness and loss of activities of daily living [3]. CIPs are often accompanied by hypermetabolism, easily leading to systemic wasting disease [4]. However, even though bone constitutes 15% of body weight and is closely linked to muscle, little attention has been paid to bone loss during and after ICU and its impact on the prognosis of this group—whether bone loss is also part of multi-organ failure? Furthermore, recent studies have uncovered multiple extra-skeletal functions of bone that are mediated by systemic crosstalk between bone-derived factors and the bone-reactive paracrine/endocrine axis [5]. Whether these functions are related to disease progression and severity in CIPs has not been established. Therefore, this review will summarize the relevant research progress in this area, hoping to help understand the changes of bone metabolism in CIPs and advance our understanding of organ dysfunction in CIPs.

## Methods

### Search summary

Studies involving critically ill patients or bone metabolism or osteoporosis were included. To find relevant original articles, we conducted a comprehensive search in the database, involving Medline through PubMed and Web of Science, and using the following words: ‘‘ critically ill patients ’’, ‘‘ OPG/RANK/RANKL ’’, ‘‘Wnt ’’, ‘‘ estrogen, ’’, ‘‘ bone metabolism ’’ and ‘‘ osteoporosis ’’ As of May 18, 2022, the papers have been searched in the language range. We also refer to the recognized literature to find other qualified research subjects. We first screened the article title and abstract, as well as publications that may involve data on bone metabolism or osteoporosis and critically ill patients.

### Inclusion and exclusion standard

This study had no national restrictions. All studies reporting on critically ill patients non-osteoporosis/osteoporosis, and laboratory confirmed osteoporotic CIPs data were included in the study. Moreover, the studies had to be limited to include raw data, be published in English and be in either abstract form or full text. Repeated studies, letters, case reports, abstracts, and comments were excluded from the study. Ninety-one relevant articles were identified.

### Causes of bone loss in CIPs

**Fig. 1:**
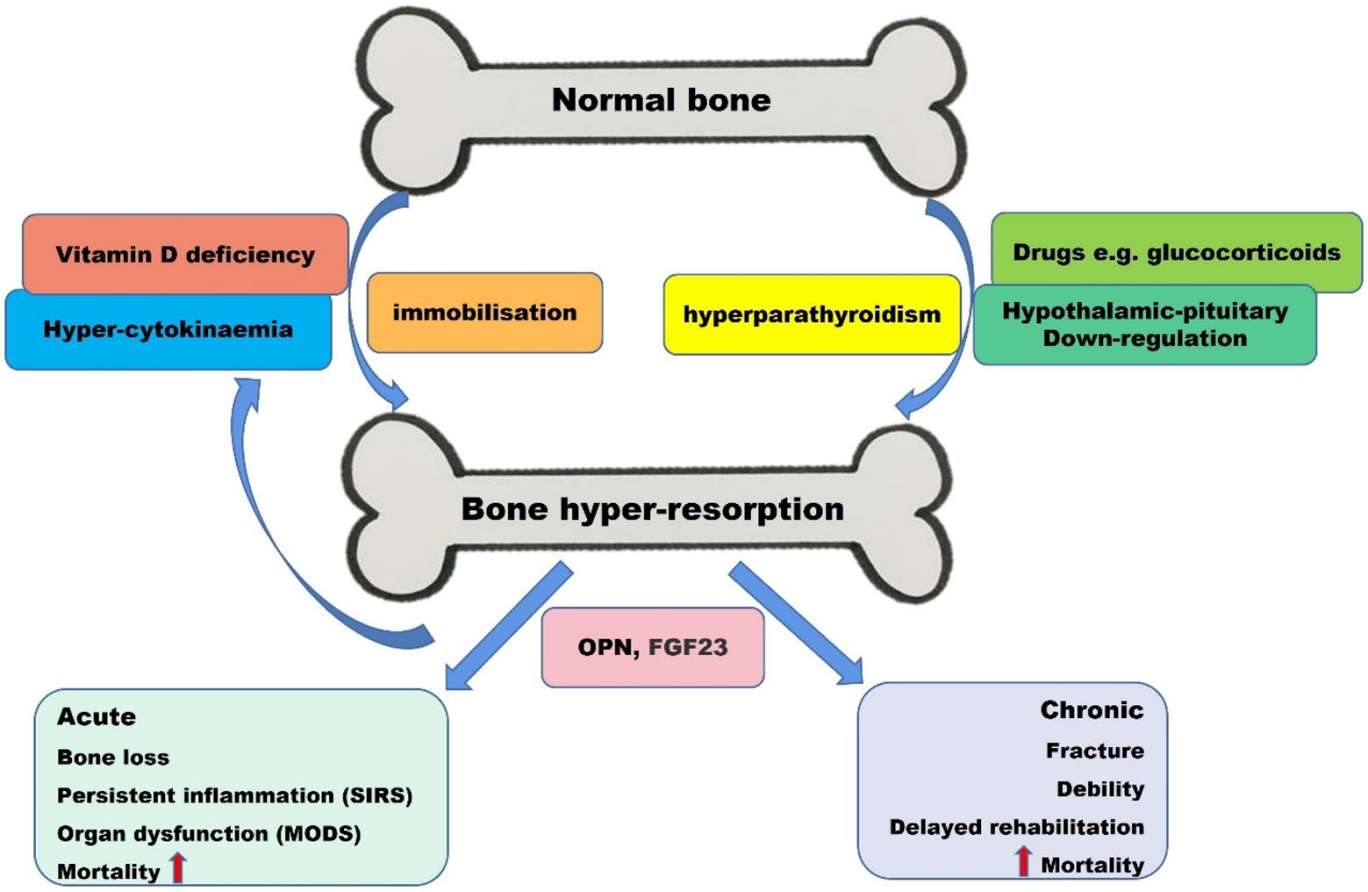
Interaction of bone hyper-resorption and critical illness

CIPs are severely compromised during ICU admission, likely independent of the original reason for ICU admission [5]. Critical illness is associated with accelerated bone loss, leading to rapid osteopenia and osteoporosis, which seriously affects CIPs’ prognosis and living quality (Fig. 1) [6]. Significant drivers of ICU-related bone loss include inflammation, neuroendocrine stress, restraint, vitamin D (VD) deficiency (especially in long-term hospitalizations), malnutrition, gut microbiota dysbiosis, and medications (corticosteroids, catecholamines, or loop diuretics). These factors increase the incidence of bone loss/osteoporosis in CIPs [4, 6].

### Detection of bone loss in CIPs

Bone condition is usually expressed by bone mineral density (BMD). The most commonly used tool for clinical detection of BMD is dual-energy X-ray absorptiometry (DXA). Although DXA is a standardized and well-validated measure, CIPs are often associated with hemodynamic instability, making the use of DXA to detect BMD in CIPs impractical. According to some published reports, computed tomography (CT) densitometry is quite even better than the DXA [7]. Chest or abdominal CT scans are available for most patients treated in the ICU, which provides CIPs with the opportunity to examine bone quality without increasing cost, radiation exposure, or taking up rescue time [8]. A previous study found that patients' femoral neck and spine BMD decreased significantly within 1 year of ICU discharge [9]. In addition, a latest study has reported that BMD of the lumbar spine was significantly reduced after ICU admission, and that non-osteoporotic patients had more significant bone loss than osteoporotic patients [7].

Bone turnover is generally increased in CIPs, decoupling between the physiological activities of osteoblasts and osteoclasts—an imbalance between bone formation and bone resorption [6, 10].

Bone turnover biomarkers (BTMs) are the products synthesized by the self-decomposition of bone tissue. Classical bone turnover markers can be divided into two categories: (1) bone resorption markers: bone tissue products secreted or metabolized by osteoclasts during bone resorption, which can reflect the activity of osteoclasts and the state of bone resorption, including hydroxyproline (HYP), pyridinoline (PYD), tartrate-resistant acid phosphatase 5b (TRAP 5b), deoxypyridinoline (DPD), the carboxyl-terminal cross-linked telopeptide of type I collagen (CTX-I), amino-terminal cross-linked telopeptide of type I collagen (NTX-I) and receptor activator for nuclear factor-κB ligand (RANKL); (2) Bone formation markers: direct or indirect products reflecting osteoblast activity and bone formation status, including bone-specific alkaline phosphatase (BSAP), osteocalcin (OC), procollagen type I N-terminal propeptide (PINP) and procollagen type I C-terminal propeptide (PICP). The serum level of BTMs can dynamically reflect the bone metabolism and detect bone loss timely. It is clinically used to monitor the progression of osteoporosis and the efficacy of anti-osteoporotic drugs [5, 11]. Thus, BTMs can also be used to assess changes in bone mass during and after critical illness.

Markers of bone resorption were 4–8 times higher than the reference range within 24 h of ICU admission and remained elevated for 1 month, suggesting that the skeletal system responds rapidly to critical illness, and bone resorption most likely begins before ICU admission. In contrast, bone formation was disproportionately inhibited. Bone formation marker levels are mainly concentrated above or within the lower limit of the normal reference range [4].

### Systemic effects of bone loss

Fibroblast growth factor 23 (FGF23), a phosphatide hormone secreted by osteocytes and osteoblasts, inhibits the activation of VD and induces excretion of Pi through proximal renal tubular epithelial cells [12]. Excessive action of FGF23 will impair bone mineralization, leading to hypophosphatemic rickets/osteomalacia. The insufficiency of FGF23 can lead to hyperphosphatemia neoplastic calcinosis with high 1.25-dihydroxyvitamin D level [13]. In addition, FGF-23 plays an essential role in regulating the expression of the OPG gene [14]. Studies have found that FGF23 is elevated during critical illness, accompanied by bone loss [15]. The loss of bone can promote glucose intolerance or systemic inflammation, which can aggravate bone loss [16]. In addition, FGF23 can also increase the risk of infection [17]. Recent studies have reported that FGF23 may be a novel target for early diagnosis of renal insufficiency and cardiovascular disease, and may also be a potential therapeutic target for patients with chronic kidney disease [18]. Some scholars even believe that elevated serum levels of FGF23 may help predict mortality and adverse neurological outcomes [19]. These suggest that FGF23 may be associated with poor outcomes and even increased mortality in CIPs [20].

Osteopontin (OPN) is an acidic secreted glycosylated phosphoprotein which originates from bone marrow hematopoietic stem cells and widely present in bone, kidney, immune system and blood system. In the skeletal system, it is secreted by osteoblasts and osteoclasts [21]. OPN is a vital regulator of inhibiting osteoblast proliferation and promoting osteoclast differentiation: on one hand, it can encourage osteoclast adhesion and improve osteoclast activity; on the other hand, osteoblasts secrete OPN after being stimulated by bone resorption stimulators (tumor necrosis factor, interleukin, etc.), while osteoclasts can interact with integrin avβ3 on the surface of OPN and adhere to bone tissue, thereby exerting an osteolytic effect [21]. OPN is a downstream signaling molecule activated by RANKL/NF-κB receptors, and the reduction of OPN secretion can trigger the reduction of bone resorption induced by PTH, RANKL and M-CSF, thereby affecting the proliferation of osteoclasts [22]. In addition, OPN also exhibits multiple immunomodulatory effects: (1) OPN is a potent neutrophil chemotactic agent; (2) OPN can upregulate the innate immune program. OPN plays a crucial role in MODS and SIRS [4]. These suggest that the serum level of OPN can not only reflect the bone metabolism level, but evaluate the severity of the disease and even the mortality in CIPs.

The development of osteoporosis is closely related to the dysfunction of three pathways: the estrogen–endocrine pathway, the Wnt/β-catenin signaling and the OPG/RANK/RANKL pathway. These three pathways have their own signal transduction targets and are closely related, forming a complex system to regulate bone metabolism in osteoporosis [23].

### The Wnt/β-catenin signaling

**Fig. 2:**
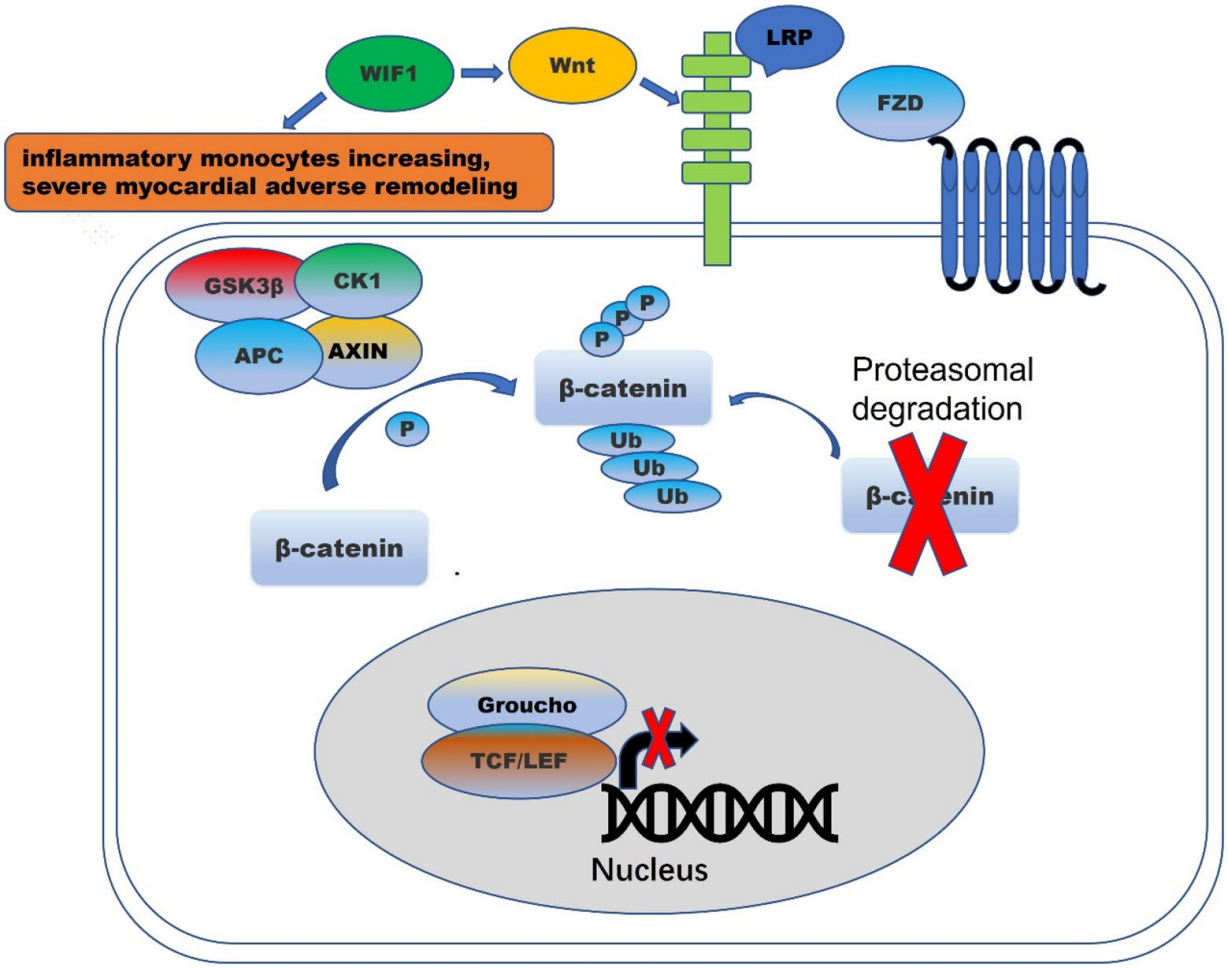
Wnt/β-catenin signaling and diseases

The Wnt/β-catenin signaling is associated with developmental processes and affects the cell cycle at different timepoints [24]. Briefly, Wnt is a growth-stimulating factor that causes cell proliferation. This pathway is activated when Wnt proteins bind to a receptor complex that includes seven transmembrane receptors of the Frizzled (Frz) family of membrane receptors and low-density lipoprotein receptor-related protein 5/6 (LRP5/6) [25]. This complex mobilizes glycogen synthase kinase 3 *β* (GSK3β) and casein kinase 1 (CK1) to the membrane and phosphorylates series on Lrp5/6, promoting the formation of semaphores, and recruits disheveled (Dvl), axis inhibition (Axin) and adenomatous polyposis Coli (APC) [26]. These will lead to the release of β-catenin, thereby increasing the intracellular concentration of β-catenin, and translocating the activated β-catenin into the nucleus, where it interacts with T cell factor (TCF)/Lymphoid enhancer (LEF) (Fig. 2). In the absence of upstream Wnt signaling, GSK3β phosphorylates residues near the amino terminus of β-catenin, hydrolyzes β-catenin via the ubiquitination pathway, and maintains β-catenin in the cytoplasm and nucleus at a lower level [27].

The Wnt/β-catenin signaling has become a hotspot in bone biology laboratories due to its importance in skeletal development, bone mass maintenance, and therapeutic potential in regenerative medicine [28]. The Wnt/β-catenin signaling is involved in cartilage, osteogenesis, muscle and adipogenesis. In addition, it plays a key role in the differentiation of the MSC lineage, affecting various aspects of skeletal development. For example, reduced expression of Lrp5 and Lrp6 in compound mutant mice can lead to limb defects [29]. Wntless, a chaperone protein required for clearance of Wnt protein secretion in the osteogenic stage, can lead to severe osteoporosis caused by the impaired bone formation and increased bone resorption [29]. β-Catenin is a crucial link in the Wnt/β-catenin signaling cascade. High levels of persistently active β-catenin inhibit mature osteoclasts and bone resorption, leading to osteosclerosis [30]. Meanwhile, blocking the Wnt/β-catenin signaling triggers the initiation of adipogenic differentiation [31]. Therefore, the stimulation of the Wnt/β-catenin signaling can promote the osteogenic differentiation of MSC lineage and inhibit its adipogenic differentiation, and the above mechanisms jointly regulate the process of osteogenesis.

Studies have shown that the Wnt/β-catenin signaling is involved in the progression of myocardial infarction, including inflammation, angiogenesis, and fibrosis [32]. Scholars found that the Wnt/β-catenin signaling was activated in cardiomyocytes located in the border region of the infarct [33]. In addition, this pathway was also activated in pro-inflammatory macrophages in the myocardial infarction area, manifested by increased levels of lymphocyte infiltration and increased expression of pro-inflammatory cytokines [34]. Another study found that loss of Wnt inhibitory factor 1 (WIF1) can lead to increased inflammatory monocytes and severe myocardial adverse remodeling, while overexpression of WIF1 impairs monocyte response and improves cardiac function [35]. During the angiogenesis stage after myocardial infarction, β-catenin accumulates in a large amount in the cytoplasm of tubular endothelial cells, thereby activating the Wnt/β-catenin signaling and inhibiting angiogenesis [36]. Myocardial fibrosis is a necessary pathophysiological process after myocardial infarction. The Wnt/β-catenin signaling plays a significant role in the regulation of cardiac fibrosis. In acute ischemic heart injury, up-regulated Wnt is first expressed in the epicardium and subsequently in cardiac fibroblasts in the injured area. Wnt induces cardiac fibroblasts to proliferate and express pro-fibrotic genes. In addition to the role of Wnt, deletion of β-catenin in cardiac fibroblasts inhibits pressure overload-induced cardiac tissue fibrosis, protects cardiac function and reduces interstitial fibrosis [36].

### The OPG/RANK/RANKL pathway

**Fig. 3:**
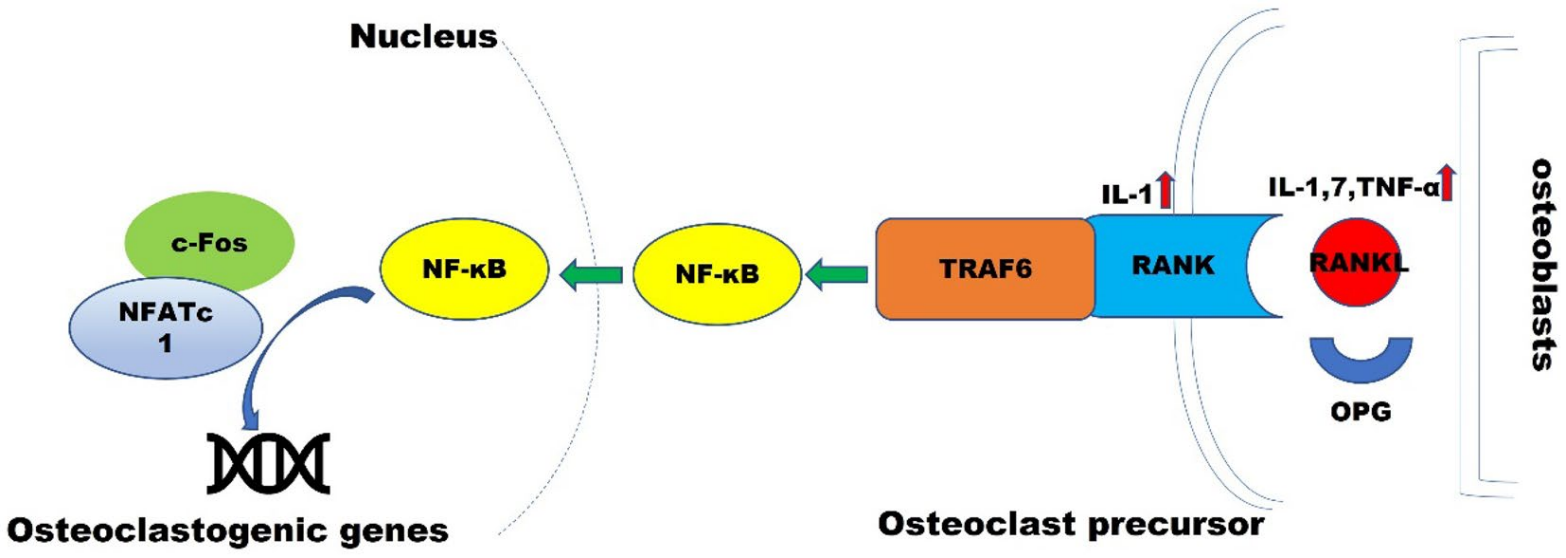
Interaction between the OPG/RANK/RANKL pathway, inflammation and immune response

The OPG/RANK/RANKL pathway is an essential pathway for regulating bone metabolism balance, regulating osteoclast activation, promoting bone resorption, and participating in the process of bone remodeling [23]. OPG, RANK, and RANKL are the members of the tumor necrosis factor-α (TNF-α) receptor superfamily. In the OPG/RANK/RANKL pathway, the competitive binding of OPG secreted by osteoblasts to RANKL inhibits bone resorption and induces apoptosis of osteoclasts (Fig. 3). In contrast the RANK receptor on the surface of osteoclasts recruits tumor necrosis factor receptor-associated factor 6 (TRAF6) by binding to RANKL and combine in cells to form trimers, and then initiate downstream cascade signaling, such as activation of nuclear factor-κB (NF-κB) [23]. Under normal physiological conditions, the two work together to maintain the balance of bone metabolism. Under pathological conditions, the "bone formation-bone resorption" coupling is disrupted, and the relationship between RANKL and OPG is dysregulated, resulting in bone loss. In addition, OPG is also expressed in mature B cells, macrophages, vascular endothelial cells, and vascular smooth muscle cells. It binds and neutralizes tumor necrosis factor-related apoptosis-inducing ligands, inhibits apoptotic bodies, and prevents atherosclerosis [37]. However, RANKL and RANK are only expressed in atherosclerotic vessel walls [38]. These suggest that the activation of the OPG/RANK/RANKL pathway is a crucial link linking osteoporosis and atherosclerosis, which may be an important factor for the increased risk of cardiovascular events in CIPs.

The OPG/RANK/RANKL pathway is considered to be associated with the regulation of inflammation and immune response, and interacts with various regulatory factors (hormones, cytokines and growth factors, etc.) [39]. Inflammatory cytokines can directly or indirectly regulate the OPG/RANK/RANKL pathway to promote or inhibit bone resorption. Interleukin-1 (IL-1) is an inflammatory cytokine that promotes bone resorption and up-regulates RANK and RANKL [40]. Interleukin-7 (IL-7) and TNF-α can only up-regulate the expression of RANKL, so they are considered as osteoclast factors, while interleukin-4 (IL-4), Interleukin-13 (IL-13) and interferon-1 can inhibit the formation of osteoclasts and are deemed to be an anti-osteoclast factor. In addition, C-reactive protein, VD, angiotensin II, etc., are all involved in regulating the OPG/RANK/RANKL pathway. In short, the OPG/RANK/RANKL pathway may be one of the major links linking disease progression to bone loss in CIPs.

### The estrogen–endocrine pathway

Estrogen receptors are highly expressed in osteoblasts, osteoclasts and osteocytes, and have protective effects on bones. Estrogen binds to the estrogen receptor, which regulates the expression of proteins encoded by estrogen target genes, such as insulin-like growth factor 1 (IGF1) and transforming growth factor-β (TGFβ) [41]. Studies have shown that estrogen can directly affects cell differentiation and apoptosis [42].

**Fig. 4:**
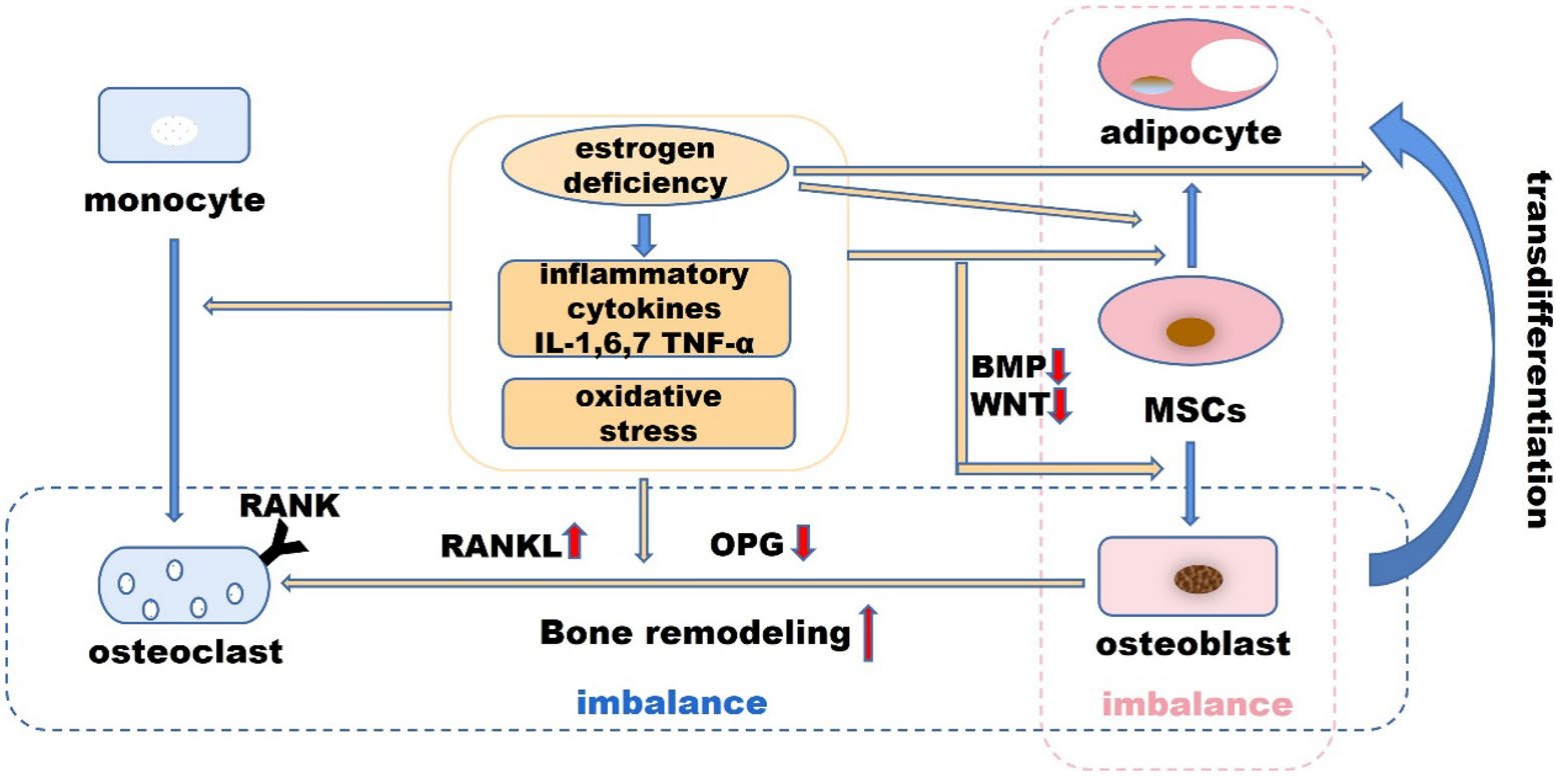
Interaction between the estrogen–endocrine pathway, the OPG/RANK/RANKL pathway and the Wnt/β-catenin signaling

The estrogen receptor complex in osteoblastic progenitor cells activates the Wnt/β-catenin signaling, manifesting as increased osteogenesis [43]. Estrogen reduces bone resorption by restraining RANKL and promoting OPG (Fig. 4). In the state of estrogen deficiency, RANKL expression increases, leading to osteoclastogenesis [44]. Osteocytes act as mechanosensors regulating bone remodeling and mineralization. In the absence of estrogen receptors and their complexes, osteocytes cannot elicit an adequate response to mechanical strain, suggesting that estrogen deficiency is associated with damage to mechanoreceptors in osteocytes [42]. Osteocytes also produce RANKL, which activates the formation of osteoclasts. Furthermore, osteocytes inhibit Wnt signaling by forming sclerostin that binds to the Wnt co-receptors LRP5/6, reducing bone formation [45]. Estrogen maintains bone stability by regulating the sclerostin production. Meanwhile, estrogen accomplishes anti-atherosclerotic effect by regulating the OPG/RANK/RANKL pathway, which can simultaneously up-regulate the expression of OPG mRNA and RANKL, ultimately prevent bone loss and atherosclerosis [46].

Animal experiments and clinical studies have shown that estrogen closely linked to immune response: increases the phagocytic function of macrophages, and shows an immunomodulatory effect with the increase of cytokines and chemokines [47–49]. Estrogen deficiency leads to the increase of IL-7 to promote the activation of T cells, and T cells secrete immunologically active substances, such as IL-1, IL-6 and TNF-α, which promote the formation of osteoclasts [42]. Estrogen deficiency also promotes T cell activation and osteoclastogenesis by increasing reactive oxygen species, leading to the production of TNF [42]. RANKL levels in mesenchymal stem cells (MSCs), T cells, and B cells are also up-regulated, leading to osteoporosis [41]. In addition, estradiol levels in septic patients are positively correlated with disease severity and mortality, regardless of gender, and the authors believe that estradiol levels can be used as prognostic markers [49].

### Administration

The impact of bone loss after critical illness on health-related costs and living quality, as well as the interaction between the skeleton and other systems, underscores the importance of an interdisciplinary comprehensive and multimodal prevention strategy, preferably in the early stages of critical illness just start.

### Nutritional support

Nutritional support for CIPs is an evolving topic. The importance of nutritional support for CIPs is increasingly recognized, especially for patients who have been hospitalized in ICU for a long time, who usually experience a severe catabolic state and require long-term maintenance of necessary nutritional support [50]. Nutritional support is the primary means of reducing the risk of malnutrition, sarcopenia, and osteoporosis in CIPs. Currently, continuous enteral nutrition is the preferred feeding method for CIPs who cannot eat on their own, and early enteral nutrition can significantly reduce the risk of death in CIPs [51], while intermittent feeding is thought to contribute to the presence of anabolic patients with the disorder restore the anabolic effects provided by amino acids [52]. However, these still need to be confirmed by clinical studies. Furthermore, the needs of every CIP are different, and their needs have not been constant throughout the process. Therefore, nutritional support should be individualized. They are well complemented by the latest guidelines of the European Society for Parenteral and Parenteral Nutrition (ESPEN) [53]. Optimizing nutrition, especially ensuring an adequate supply of amino acids, during ICU stay and after discharge, can synergize with early risers and drug therapy to increase the rate of protein synthesis in muscle [54]. Special attention should also be paid to the intake of minerals, such as calcium [55] and selenium [56]. There are no clinical data on the effects of protein- and mineral-adjusted nutritional strategies on bone loss and bone recovery in CIPs.

### Vitamin D

The prevalence of VD deficiency in ICU is usually between 40% and 70% [57]. Numbers of observational studies have consistently demonstrated an association between low VD levels and poor clinical outcomes in CIPs [57]. The beneficial effects of VD on the musculoskeletal system are beyond doubt. Relevant guidelines point out that most people need regular VD supplementation, and daily supplementation of 600–2000 IU of VD can maintain normal VD levels [58]. For CIPs, VD-related side effects are rare, and there are no reports of VD intoxication. However, identifying adverse events in CIPs is difficult due to the complexity of treatment and underlying diseases. Relevant studies have primarily used oral cholecalciferol in doses ranging from 200 IU to 540,000 IU, with limited reported side effects [59–62]. In the VITDAL–ICU study, only 1% of patients developed mild hypercalcemia, but these patients were asymptomatic. There were no significant differences in calcium, phosphorus, and renal parameters between the two groups in this trial [59]. In addition, the patients in these studies had VD levels well below the toxic dose (200 ng/mL). Some scholars believe that these patients are at increased risk of fractures and falls, but the available evidence for critical illness in the VITdAL–ICU trial does not suggest an increased risk of falls or fractures [59]. Research on the potential effects of different doses of VD on ICU mortality is ongoing [57, 63, 64].

### Gut microbiome regulation

The microbiome affects bone conditions [65]. Experimental data suggest that the use of probiotics to modulate the gut microbiota can increase bone mass [66]. Administration of prebiotics leads to enhanced calcium absorption and favorable changes in gut microbiota composition, resulting in improved bone mass [67]. In addition, a variety of endogenous and iatrogenic factors, such as gastrointestinal motility disorders, changes in intraluminal pH, increased catecholamine production, antibiotic therapy, proton pump inhibitors, opioids, and enteral feeding, can contribute to the development of severe disruption of the microbiota in CIPs [68]. Thus, microbiome modulation may become a novel adjuvant for the prevention and even treatment of ICU-related osteoporosis or osteoporosis.

### Recovery treatment

Clinical data suggest that early mobilization is feasible and well-tolerated in most CIPs [69, 70]. Multiple meta-analyses have shown that early mobilization improves physical performance [71–73]. Research has found that whole-body vibration is an effective training method for increasing BMD [74]. There has been no study of the effect of physical rehabilitation on bone mass and fracture risk in patients during their ICU stay. However, physical rehabilitation is an extremely vital part of ICU clinical practice guidelines [75, 76]. Therefore, it is necessary to maintain rehabilitation training in the ICU, transfer from ICU to the general ward, and after discharge [77].

### Bone-promoting drugs

Hypermetabolism frequently occurs in CIPs, which can easily lead to systemic wasting diseases. In addition, the skeletal system is difficult to escape. Therefore, some scholars have started to study “the effect of regulating bone metabolism on systemic metabolism’’. Androgens are a classic drug that promotes bone formation. The synthetic testosterone analog oxyandrosterone may be a potential therapeutic option. A recent meta-analysis showed that oxyandrosterone increased BMD without affecting mortality in severely burned patients [78]. In addition, oxyandrosterone can reduce weight loss in trauma patients [79].

Teriparatide, a recombinant human parathyroid hormone (PTH), is one of the most effective anabolic therapies for the severe osteoporosis. Intermittent skeletal exposure to PTH increases bone formation with a more minor increase in bone resorption compared to continuous exposure, thus exhibiting a net anabolic effect [80]. These culminated in a pivotal clinical trial, where teriparatide at 20 μg/day (the FDA-approved dose) reduced the risk of vertebral fracture by 65% (RR 0.35, 95% CI 0.22–0.55), and reduced the risk of nonvertebral fractures by 53% (RR 0.47, 95% CI 0.25–0.88) [80]. Because high doses of teriparatide can increase the risk of osteosarcoma in growing rodents, the FDA limited the duration of clinical treatment with teriparatide to 24 months [81]. However, in subsequent follow-up, it was found that the risk of osteosarcoma in patients treated with teriparatide was not significantly higher than that in the general population [82]. Regrettably, there are no data on PTH in CIPs, but antiresorptive therapy is biologically more appropriate to reduce the risk of fractures associated with critical illness.

### Anti-bone resorption drugs

The loss of BMD was significant in CIPs receiving anti-osteoporosis drugs compared with patients not receiving anti-osteoporosis drugs [83]. However, all anti-osteoporosis drugs should be started after maintaining adequate VD levels to reduce the risk of severe hypocalcemia.

Bisphosphonates can specifically combine with hydroxyapatite in bone to inhibit osteoclast activity, and ultimately reduce bone resorption. It is the most widely used clinical drug for the prevention and treatment of osteoporosis. Compared with CIPs who did not receive bisphosphonates, CIPs who received bisphosphonates had significantly lower BMD loss, combined bisphosphonates with VD had a better prognosis and lower mortality [84]. Moreover, we should consider the contraindications and potential side effects of bisphosphonates, such as hypocalcemia, renal impairment and atrial fibrillation, which may also limit the use of bisphosphonates in CIPs.

Denosumab is a bone resorption inhibitor with a unique mechanism, which specifically targets RANKL, inhibits the activation and development of osteoclasts, reduces bone resorption and increases BMD. In a 1-year study, denosumab was more effective against osteoporosis than bisphosphonates. Unfortunately, rebound vertebral fractures are prone to occur after the interruption of denosumab [85]. However, patients treated with denosumab for less than 2 years had significantly lower rates of rebound vertebral fractures compared with patients treated with long-term denosumab [86]. This suggests that the rebound phenomenon may not occur with a single dose during ICU stay. Denosumab has been shown to improve bone metabolism in patients with spinal cord injury [87]. However, studies on the efficacy of denosumab in CIPs are lacking.

Research data suggest that monoclonal antibody inhibitors or deficiency of sclerostin can enhance bone strength [88]. Therefore, sclerostin antibodies such as romosuzumab (AMG-785) [89] and BPS804 [90] have been gradually included in clinical trials. Results of a phase 2 study comparing the anti-osteoporotic efficacy of 12 month romosuzumab with placebo, alendronate, and teriparatide in postmenopausal women, showed that the romosuzumab group’s lumbar spine BMD increased by 11.3%, and hip BMD increased by 4.1%, which were significantly higher than other groups [91]. It is inspiring that romosuzumab has been approved in Europe to treat severe osteoporosis in postmenopausal women with a high risk of fractures [5]. Unfortunately, there are no data on efficacy in CIPs.

## Conclusions

The skeletal system is closely associated with the immune system, cardiovascular system and other systems through the above three pathways and self-secreted factors. Critical illness has a long-term impact on bone metabolism, and changes in bone metabolism will also affect other systems through the above multiple pathways. Accordingly, scholars even put forward the hypothesis of bone failure—the rapid loss of bone in CIPs is an unrecognized component of MODS/ICU wasting [4]. If the hypothesis holds, it will not only advance our understanding of ICU organ dysfunction and systemic inflammation, but also provide new therapeutic targets for critical illness.

## Data Availability

Not applicable.

## References

[1] Goossens C, Marques MB, Derde S, Vander Perre S, Dufour T, Thiessen SE, Guiza F, Janssens T, Hermans G, Vanhorebeek I (2017). Premorbid obesity, but not nutrition, prevents critical illness-induced muscle wasting and weakness. J Cachexia Sarcopenia Muscle.

[2] Benz F, Roy S, Trautwein C, Roderburg C, Luedde T (2016). Circulating microRNAs as biomarkers for sepsis. Int J Mol Sci.

[3] Yang F, Fang F (2021). Research progress in diagnosis and evaluation of intensive care unit-acquired weakness. Int J Mol Sci.

[4] Lee P, Nair P, Eisman JA, Center JR (2016). Bone failure in critical illness. Crit Care Med.

[5] Rousseau AF, Kerschan-Schindl K, Scherkl M, Amrein K (2020). Bone metabolism and fracture risk during and after critical illness. Curr Opin Crit Care.

[6] Orford N, Cattigan C, Brennan SL, Kotowicz M, Pasco J, Cooper DJ (2014). The association between critical illness and changes in bone turnover in adults: a systematic review. Osteoporos Int.

[7] Hongo T, Kotake K, Muramatsu H, Omura D, Yano Y, Hasegawa D, Momoki N, Takahashi K, Nozaki S, Fujiwara T (2019). Loss of bone mineral density following sepsis using Hounsfield units by computed tomography. Acute Med Surg.

[8] Jang S, Graffy PM, Ziemlewicz TJ, Lee SJ, Summers RM, Pickhardt PJ (2019). Opportunistic osteoporosis screening at routine abdominal and thoracic ct: normative l1 trabecular attenuation values in more than 20 000 adults. Radiology.

[9] Orford NR, Lane SE, Bailey M, Pasco JA, Cattigan C, Elderkin T, Brennan-Olsen SL, Bellomo R, Cooper DJ, Kotowicz MA (2016). Changes in bone mineral density in the year after critical illness. Am J Respir Crit Care Med.

[10] Orford NR, Pasco JA, Kotowicz MA (2019). Osteoporosis and the critically ill patient. Crit Care Clin.

[11] Vasikaran SD, Miura M, Pikner R, Bhattoa HP, Cavalier E (2021). Metabolism I-IJCoB: practical considerations for the clinical application of bone turnover markers in osteoporosis. Calcif Tissue Int.

[12] Wei X, Huang X, Liu N, Qi B, Fang S, Zhang Y (2021). Understanding the stony bridge between osteoporosis and vascular calcification: impact of the FGF23/Klotho axis. Oxid Med Cell Longev.

[13] Bar L, Stournaras C, Lang F, Foller M (2019). Regulation of fibroblast growth factor 23 (FGF23) in health and disease. FEBS Lett.

[14] Nakahara T, Kawai-Kowase K, Matsui H, Sunaga H, Utsugi T, Iso T, Arai M, Tomono S, Kurabayashi M (2016). Fibroblast growth factor 23 inhibits osteoblastic gene expression and induces osteoprotegerin in vascular smooth muscle cells. Atherosclerosis.

[15] Fukao W, Hasuike Y, Yamakawa T, Toyoda K, Aichi M, Masachika S, Kantou M, Takahishi SI, Iwasaki T, Yahiro M (2018). Oral versus intravenous iron supplementation for the treatment of iron deficiency anemia in patients on maintenance hemodialysis-effect on fibroblast growth factor-23 metabolism. J Ren Nutr.

[16] Klein GL (2019). The role of the musculoskeletal system in post-burn hypermetabolism. Metabolism.

[17] Schnedl C, Fahrleitner-Pammer A, Pietschmann P, Amrein K (2015). FGF23 in acute and chronic illness. Dis Markers.

[18] Lu X, Hu MC (2017). Klotho/FGF23 axis in chronic kidney disease and cardiovascular disease. Kidney Dis.

[19] Spaich S, Zelniker T, Endres P, Stiepak J, Uhlmann L, Bekeredjian R, Chorianopoulos E, Giannitsis E, Backs J, Katus HA (2016). Fibroblast growth factor 23 (FGF-23) is an early predictor of mortality in patients with cardiac arrest. Resuscitation.

[20] Leaf DE, Siew ED, Eisenga MF, Singh K, Mc Causland FR, Srivastava A, Ikizler TA, Ware LB, Ginde AA, Kellum JA (2018). Fibroblast growth factor 23 associates with death in critically ill patients. Clin J Am Soc Nephrol.

[21] Icer MA, Gezmen-Karadag M (2018). The multiple functions and mechanisms of osteopontin. Clin Biochem.

[22] Si J, Wang C, Zhang D, Wang B, Zhou Y (2020). Osteopontin in bone metabolism and bone diseases. Med Sci Monit.

[23] Hu H, He X, Zhang Y, Wu R, Chen J, Lin Y, Shen B (2020). MicroRNA alterations for diagnosis, prognosis, and treatment of osteoporosis: a comprehensive review and computational functional survey. Front Genet.

[24] Nusse R, Clevers H (2017). Wnt/beta-catenin signaling, disease, and emerging therapeutic modalities. Cell.

[25] Joiner DM, Ke J, Zhong Z, Xu HE, Williams BO (2013). LRP5 and LRP6 in development and disease. Trends Endocrinol Metab.

[26] Niehrs C, Shen J (2010). Regulation of Lrp6 phosphorylation. Cell Mol Life Sci.

[27] Kestler HA, Kuhl M (2008). From individual Wnt pathways towards a wnt signalling network. Philos Trans R Soc Lond B Biol Sci.

[28] Monroe DG, McGee-Lawrence ME, Oursler MJ, Westendorf JJ (2012). Update on wnt signaling in bone cell biology and bone disease. Gene.

[29] Baron R, Kneissel M (2013). WNT signaling in bone homeostasis and disease: from human mutations to treatments. Nat Med.

[30] Wei W, Zeve D, Suh JM, Wang X, Du Y, Zerwekh JE, Dechow PC, Graff JM, Wan Y (2011). Biphasic and dosage-dependent regulation of osteoclastogenesis by beta-catenin. Mol Cell Biol.

[31] Bennett CN, Ross SE, Longo KA, Bajnok L, Hemati N, Johnson KW, Harrison SD, MacDougald OA (2002). Regulation of wnt signaling during adipogenesis. J Biol Chem.

[32] Fu WB, Wang WE, Zeng CY (2019). Wnt signaling pathways in myocardial infarction and the therapeutic effects of Wnt pathway inhibitors. Acta Pharmacol Sin.

[33] Oerlemans MI, Goumans MJ, van Middelaar B, Clevers H, Doevendans PA, Sluijter JP (2010). Active Wnt signaling in response to cardiac injury. Basic Res Cardiol.

[34] Huang L, Xiang M, Ye P, Zhou W, Chen M (2018). Beta-catenin promotes macrophage-mediated acute inflammatory response after myocardial infarction. Immunol Cell Biol.

[35] Meyer IS, Jungmann A, Dieterich C, Zhang M, Lasitschka F, Werkmeister S, Haas J, Muller OJ, Boutros M, Nahrendorf M (2017). The cardiac microenvironment uses non-canonical WNT signaling to activate monocytes after myocardial infarction. EMBO Mol Med.

[36] Zhang Q, Wang L, Wang S, Cheng H, Xu L, Pei G, Wang Y, Fu C, Jiang Y, He C (2022). Signaling pathways and targeted therapy for myocardial infarction. Signal Transduct Target Ther.

[37] Perez de Ciriza C, Lawrie A, Varo N (2015). Osteoprotegerin in cardiometabolic disorders. Int J Endocrinol.

[38] Rochette L, Meloux A, Rigal E, Zeller M, Cottin Y, Vergely C (2018). The role of osteoprotegerin in the crosstalk between vessels and bone: Its potential utility as a marker of cardiometabolic diseases. Pharmacol Ther.

[39] Ohigashi I, Nitta T, Lkhagvasuren E, Yasuda H, Takahama Y (2011). Effects of RANKL on the thymic medulla. Eur J Immunol.

[40] Zupan J, Jeras M, Marc J (2013). Osteoimmunology and the influence of pro-inflammatory cytokines on osteoclasts. Biochem Med.

[41] Black DM, Rosen CJ (2016). Clinical practice postmenopausal osteoporosis. N Engl J Med.

[42] Cheng CH, Chen LR, Chen KH (2022). Osteoporosis due to hormone imbalance: an overview of the effects of estrogen deficiency and glucocorticoid overuse on bone turnover. Int J Mol Sci.

[43] Almeida M, Iyer S, Martin-Millan M, Bartell SM, Han L, Ambrogini E, Onal M, Xiong J, Weinstein RS, Jilka RL (2013). Estrogen receptor-alpha signaling in osteoblast progenitors stimulates cortical bone accrual. J Clin Invest.

[44] Abu-Amer Y (2013). NF-kappaB signaling and bone resorption. Osteoporos Int.

[45] Bado I, Gugala Z, Fuqua SAW, Zhang XH (2017). Estrogen receptors in breast and bone: from virtue of remodeling to vileness of metastasis. Oncogene.

[46] Ma B, Liu J, Zhang Q, Ying H, Sun AJ, Wu J, Wang D, Li Y, Liu JY (2013). Metabolomic profiles delineate signature metabolic shifts during estrogen deficiency-induced bone loss in rat by GC-TOF/MS. PLoS ONE.

[47] Chakraborty B, Byemerwa J, Shepherd J, Haines CN, Baldi R, Gong W, Liu W, Mukherjee D, Artham S, Lim F (2021). Inhibition of estrogen signaling in myeloid cells increases tumor immunity in melanoma. J Clin Invest.

[48] Kovats S (2015). Estrogen receptors regulate innate immune cells and signaling pathways. Cell Immunol.

[49] Findikli HA, Erdogan M (2021). Serum G protein-coupled estrogen receptor-1 levels and its relation with death in patients with sepsis: a prospective study. Minerva Anestesiol.

[50] Barazzoni R, Bischoff SC, Breda J, Wickramasinghe K, Krznaric Z, Nitzan D, Pirlich M, Singer P (2020). endorsed by the EC: ESPEN expert statements and practical guidance for nutritional management of individuals with SARS-CoV-2 infection. Clin Nutr.

[51] Ojo O, Ojo OO, Feng Q, Boateng J, Wang X, Brooke J, Adegboye ARA (2022). The effects of enteral nutrition in critically ill patients with COVID-19: a systematic review and meta-analysis. Nutrients.

[52] Bear DE, Hart N, Puthucheary Z (2018). Continuous or intermittent feeding: pros and cons. Curr Opin Crit Care.

[53] Singer P, Blaser AR, Berger MM, Alhazzani W, Calder PC, Casaer MP, Hiesmayr M, Mayer K, Montejo JC, Pichard C (2019). ESPEN guideline on clinical nutrition in the intensive care unit. Clin Nutr.

[54] Wolfe RR (2018). The 2017 sir david p cuthbertson lecture amino acids and muscle protein metabolism in critical care. Clin Nutr.

[55] Rizzoli R (2014). Nutritional aspects of bone health. Best Pract Res Clin Endocrinol Metab.

[56] van Dronkelaar C, van Velzen A, Abdelrazek M, van der Steen A, Tieland WPJM, M,  (2018). Minerals and sarcopenia the role of calcium iron magnesium phosphorus potassium selenium sodium and zinc on muscle mass muscle strength and physical performance in older adults a systematic review. J Am Med Dir Assoc.

[57] Amrein K, Papinutti A, Mathew E, Vila G, Parekh D (2018). Vitamin D and critical illness: what endocrinology can learn from intensive care and vice versa. Endocr Connect.

[58] Holick MF, Binkley NC, Bischoff-Ferrari HA, Gordon CM, Hanley DA, Heaney RP, Murad MH, Weaver CM, Endocrine S (2011). Evaluation, treatment, and prevention of vitamin D deficiency: an endocrine society clinical practice guideline. J Clin Endocrinol Metab.

[59] Amrein K, Schnedl C, Holl A, Riedl R, Christopher KB, Pachler C, Urbanic Purkart T, Waltensdorfer A, Munch A, Warnkross H (2014). Effect of high-dose vitamin D3 on hospital length of stay in critically ill patients with vitamin D deficiency: the VITdAL-ICU randomized clinical trial. JAMA.

[60] Quraishi SA, De Pascale G, Needleman JS, Nakazawa H, Kaneki M, Bajwa EK, Camargo CA, Bhan I (2015). Effect of cholecalciferol supplementation on vitamin D status and cathelicidin levels in sepsis: a randomized Placebo-Controlled Trial. Crit Care Med.

[61] Mata-Granados JM, Vargas-Vasserot J, Ferreiro-Vera C (2010). Luque de Castro MD, Pavon RG, Quesada Gomez JM: Evaluation of vitamin D endocrine system (VDES) status and response to treatment of patients in intensive care units (ICUs) using an on-line SPE-LC-MS/MS method. J Steroid Biochem Mol Biol.

[62] Nair P, Venkatesh B, Lee P, Kerr S, Hoechter DJ, Dimeski G, Grice J, Myburgh J, Center JR (2015). A randomized study of a single dose of intramuscular cholecalciferol in critically Ill adults. Crit Care Med.

[63] National Heart L, Blood Institute PCTN, Ginde AA, Brower RG, Caterino JM, Finck L, Banner-Goodspeed VM, Grissom CK, Hayden D, Hough CL (2019). Early high-dose vitamin D3 for critically Ill, vitamin D-deficient patients. N Engl J Med.

[64] Amrein K, Parekh D, Westphal S, Preiser JC, Berghold A, Riedl R, Eller P, Schellongowski P, Thickett D, Meybohm P (2019). Effect of high-dose vitamin D3 on 28-day mortality in adult critically ill patients with severe vitamin D deficiency: a study protocol of a multicentre, placebo-controlled double-blind phase III RCT (the VITDALIZE study). BMJ Open.

[65] Hernandez CJ, Guss JD, Luna M, Goldring SR (2016). Links between the microbiome and bone. J Bone Miner Res.

[66] D'Amelio P, Sassi F (2018). Gut microbiota, immune system, and bone. Calcif Tissue Int.

[67] Whisner CM, Castillo LF (2018). Prebiotics, bone and mineral metabolism. Calcif Tissue Int.

[68] Schuurman AR, Kullberg RFJ, Wiersinga WJ (2022). Probiotics in the intensive care unit. Antibiotics.

[69] Hruska P (2016). Early mobilization of mechanically ventilated patients. Crit Care Nurs Clin North Am.

[70] Mayer KP, Hornsby AR, Soriano VO, Lin TC, Cunningham JT, Yuan H, Hauschild CE, Morris PE, Neyra JA (2020). Safety, feasibility, and efficacy of early rehabilitation in patients requiring continuous renal replacement: a quality improvement study. Kidney Int Rep.

[71] Zhang L, Hu W, Cai Z, Liu J, Wu J, Deng Y, Yu K, Chen X, Zhu L, Ma J (2019). Early mobilization of critically ill patients in the intensive care unit: a systematic review and meta-analysis. PLoS ONE.

[72] Fuke R, Hifumi T, Kondo Y, Hatakeyama J, Takei T, Yamakawa K, Inoue S, Nishida O (2018). Early rehabilitation to prevent postintensive care syndrome in patients with critical illness: a systematic review and meta-analysis. BMJ Open.

[73] Connolly B, O'Neill B, Salisbury L, Blackwood B (2016). Enhanced recovery after critical illness programme g: physical rehabilitation interventions for adult patients during critical illness: an overview of systematic reviews. Thorax.

[74] Bemben D, Stark C, Taiar R, Bernardo-Filho M (2018). Relevance of whole-body vibration exercises on muscle strength/power and bone of elderly individuals. Dose Response.

[75] Vincent JL, Shehabi Y, Walsh TS, Pandharipande PP, Ball JA, Spronk P, Longrois D, Strom T, Conti G, Funk GC (2016). Comfort and patient-centred care without excessive sedation: the eCASH concept. Intensive Care Med.

[76] Devlin JW, Skrobik Y, Gelinas C, Needham DM, Slooter AJC, Pandharipande PP, Watson PL, Weinhouse GL, Nunnally ME, Rochwerg B (2018). Clinical practice guidelines for the prevention and management of pain, agitation/sedation, delirium, immobility, and sleep disruption in adult patients in the ICU. Crit Care Med.

[77] Major ME, Kwakman R, Kho ME, Connolly B, McWilliams D, Denehy L, Hanekom S, Patman S, Gosselink R, Jones C (2016). Surviving critical illness: what is next? An expert consensus statement on physical rehabilitation after hospital discharge. Crit Care.

[78] Ring J, Heinelt M, Sharma S, Letourneau S, Jeschke MG (2020). Oxandrolone in the treatment of burn injuries: a systematic review and meta-analysis. J Burn Care Res.

[79] Asehnoune K, Vourc'h M, Roquilly A (2019). Hormone therapy in trauma patients. Crit Care Clin.

[80] Khosla S, Hofbauer LC (2017). Osteoporosis treatment: recent developments and ongoing challenges. Lancet Diabetes Endocrinol.

[81] Vahle JL, Long GG, Sandusky G, Westmore M, Ma YL, Sato M (2004). Bone neoplasms in F344 rats given teriparatide [rhPTH(1–34)] are dependent on duration of treatment and dose. Toxicol Pathol.

[82] Andrews EB, Gilsenan AW, Midkiff K, Sherrill B, Wu Y, Mann BH, Masica D (2012). The US postmarketing surveillance study of adult osteosarcoma and teriparatide: study design and findings from the first 7 years. J Bone Miner Res.

[83] Orford NR, Bailey M, Bellomo R, Pasco JA, Cattigan C, Elderkin T, Brennan-Olsen SL, Cooper DJ, Kotowicz MA (2017). The association of time and medications with changes in bone mineral density in the 2 years after critical illness. Crit Care.

[84] Lee P, Ng C, Slattery A, Nair P, Eisman JA, Center JR (2016). Preadmission bisphosphonate and mortality in critically ill patients. J Clin Endocrinol Metab.

[85] Miller PD, Pannacciulli N, Brown JP, Czerwinski E, Nedergaard BS, Bolognese MA, Malouf J, Bone HG, Reginster JY, Singer A (2016). Denosumab or zoledronic acid in postmenopausal women with osteoporosis previously treated with oral bisphosphonates. J Clin Endocrinol Metab.

[86] Anastasilakis AD, Polyzos SA, Makras P, Aubry-Rozier B, Kaouri S, Lamy O (2017). Clinical features of 24 patients with rebound-associated vertebral fractures after denosumab discontinuation: systematic review and additional cases. J Bone Miner Res.

[87] Gifre L, Ruiz-Gaspa S, Carrasco JL, Portell E, Vidal J, Muxi A, Monegal A, Guanabens N, Peris P (2017). Effect of recent spinal cord injury on the OPG/RANKL system and its relationship with bone loss and the response to denosumab therapy. Osteoporos Int.

[88] Li X, Ominsky MS, Niu QT, Sun N, Daugherty B, D'Agostin D, Kurahara C, Gao Y, Cao J, Gong J (2008). Targeted deletion of the sclerostin gene in mice results in increased bone formation and bone strength. J Bone Miner Res.

[89] Lim SY, Bolster MB (2017). Profile of romosozumab and its potential in the management of osteoporosis. Drug Des Devel Ther.

[90] Lewiecki EM (2014). Role of sclerostin in bone and cartilage and its potential as a therapeutic target in bone diseases. Ther Adv Musculoskelet Dis.

[91] McClung MR, Grauer A, Boonen S, Bolognese MA, Brown JP, Diez-Perez A, Langdahl BL, Reginster JY, Zanchetta JR, Wasserman SM (2014). Romosozumab in postmenopausal women with low bone mineral density. N Engl J Med.

